# Reply to: ‘Browning capabilities of human primary adipose-derived stromal cells compared to SGBS cells’

**DOI:** 10.1038/s41598-020-64706-w

**Published:** 2020-06-15

**Authors:** Chia Rou Yeo, Madhur Agrawal, Shawn Hoon, Asim Shabbir, Manu Kunaal Shrivastava, Shiqi Huang, Chin Meng Khoo, Vanna Chhay, Muhammad Shabeer, E Shyong Tai, Antonio Vidal-Puig, Sue-Anne Toh

**Affiliations:** 1 Department of Medicine, Yong Loo Lin School of Medicine, National University of Singapore, 117599 Singapore, Singapore; 2 Molecular Engineering Laboratory, Biomedical Sciences Institutes, A*Star, 138668 Singapore, Singapore; 3 Department of Surgery, National University Hospital, 119074 Singapore, Singapore; 4 Metabolic Research Laboratories, Institute of Metabolic Science, Addenbrooke’s Hospital, University of Cambridge, Cambridge, CB2 0QC UK; 5 Food Science and Technology Program, Department of Chemistry, National University of Singapore, 117542 Singapore, Singapore; 6 Wellcome Trust Sanger Institute, Wellcome Trust Genome Campus, Hinxton, Cambridge, CB10 1SA UK

**Keywords:** Cell signalling, Transcriptomics

**replying to**: D. Halbgebauer et al.; *Scientific Reports* 10.1038/s41598-020-64369-7 (2020).

## Introduction

Pharmacological browning/beiging of adipose tissue has the potential to be an important therapeutic strategy to combat obesity and associated co-morbidities. SGBS adipocytes are non-transformed primary human adipocytes with an ability to grow and differentiate up to 50 passages. Due to extended growth capacity when compared to other available primary human adipocytes, SGBS adipocytes are commonly used as a model for human adipose in *in-vitro* studies. Tews *et al*. reported equal browning capacity in SGBS adipocytes when compared with primary human adipocytes derived from the mammary region. Results in the manuscript appear to be discordant from findings in our previously published Scientific Reports paper: “SGBS cells as a model of human adipocyte browning: A comprehensive comparative study with primary human white subcutaneous adipocytes”. We observed a more robust browning signature in SGBS adipocytes as compared to primary human adipocytes derived from human subcutaneous abdominal adipose tissue. Here, we discuss the key differences between study design and methods used in both studies, which may have led to the differences in the browning phenotype observed. Together, observations and inferences from both studies support the presence of functional heterogeneity of different adipose tissue depots in humans. Collectively, our efforts provide insights into considerations for choosing appropriate cell culture models and experimental conditions to be used in future studies related to human adipose tissue browning.

Halbgebauer *et al*. used different pro-adipogenic stimuli (rosiglitazone vs indomethacin) and compared SGBS adipocytes to primary human ASCs (adipose stem/stromal cells), derived from mammary glands, during and post differentiation. Both stimuli are commonly used as supplements in pre-adipocyte differentiation medium *in-vitro*^1^. Rosiglitazone is a PPARgamma agonist and indomethacin is a non-steroidal anti-inflammatory drug. Compared to indomethacin, the presence of rosiglitazone increased differentiation capacity, gene and protein expression of browning markers including UCP1, and oxygen consumption rate (OCR) in both adipocyte cell types. When differentiated with medium containing rosiglitazone, UCP1 expression was higher in SGBS adipocytes whereas PGC1α and DIO2 expression were higher in ASCs. Functionally, proton leak, basal respiration and maximal respiration were higher in ASCs compared to SGBS adipocytes, suggesting that ASCs and SGBS bear similar browning capacity.

In our experiments, SGBS cells were differentiated in medium containing rosiglitazone^2,3^ and compared to subcutaneous adipose tissue derived ASCs (referred to as primary human white subcutaneous [PHWSC] in this response and in our manuscript) with medium containing indomethacin (purchased from Lonza)^4^. Under these conditions, both cell types had a similar morphology and we also observed a similar percentage of differentiated adipocytes. Post differentiation, OCR, proton leak and maximal respiration were significantly higher in SGBS adipocytes as compared to PHWSCs. In both studies, PGC1α and DIO2 expression was higher in ASCs as compared to SGBS adipocytes. Here, we discuss different features of the experimental design between the two studies, which may have resulted in differences in results, and the implications on the interpretation of the findings.

### Differences in the site of adipose explant collection and clinical characteristics of donors

PHWSC donors in our study were obese Asians and adipose tissue samples were collected from the abdominal region from individuals undergoing bariatric surgery; whereas Tews *et al*. obtained explants from mammary region from females undergoing elective surgery as well as deep neck and subcutaneous neck adipose tissue from patients undergoing neck surgery. For the latter, these adipose tissue samples were excised from patients undergoing neck surgery for malignancies or nodular goitre^5^. It is important to note that adipose tissue from the neck region are inherently different from most other adipose depots^6–8^. The neck region is primarily where the browning mechanism was disclosed and beside the UCP1 positive cells, it contains at least two types of adipocytes originating from two distinct mesenchymal stem cell lineages. The classic brown adipocytes with precursors in common with muscle cells and newly recognized beige adipocytes which have the same precursor as white adipose cells^7^. This unique aspect of neck adipose tissue should be should be taken into consideration when interpreting the results.

For *in-vitro* cell culture experiments, Tews *et al*. isolated adipocytes by collagenase digestion of mammary region adipose tissue. Adipocytes derived from the mammary fat pad function differently from those in other fat depots^9,10^. For example, trans-differentiation of subcutaneous white adipocytes derived from mouse mammary gland produce “pink adipocytes”. Pink adipocytes are believed to arise exclusively in female subcutaneous depots during pregnancy and lactation^10^.

Other factors such as BMI, can have a negative impact on the differentiation capacity of ASCs derived from mammary adipose tissue^11^. In ASCs derived from human subcutaneous adipose tissue depots, a donor with an obese phenotype is associated with reduced capacity of their adipocytes to display characteristics of browning, as measured by UCP1 protein expression^12^. Compared to adipocytes derived from lean individuals, UCP1 protein expression was close to zero in differentiated adipocytes from obese individuals, irrespective of similar differentiation capacity. Even in healthy men, body fat percentage and BMI have been shown to have a negative impact on functional brown adipose tissue^13^.

The SGBS adipocytes used in our study were derived from a 3-month old infant^2^, whereas Tews *et al*. reported using SGBS adipocytes derived from a 5-month old infant. Considering the disease heterogeneity of the SGBS syndrome^14^, cells derived from different donors could also potentially display varying phenotypes *in-vitro*.

### Differentiation of SGBS preadipocytes or hASCs in the presence of indomethacin or rosiglitazone in our experiments

**Figure 1:**
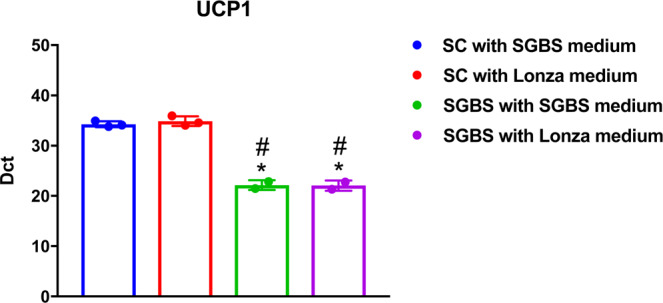
Differentiation of PHWSC and SGBS preadipocytes in both SGBS and Lonza medium, respectively. To rule out the possibility of UCP1 expression being induced by differing media compositions, SGBS (n = 2 in duplicates) and SC (n = 3 in duplicates) were differentiated *in vitro* using both sets of media, on two separate occasions. Statistical differences were analysed by Kruskal–Wallis one-way ANOVA followed by Dunn’s test for multiple comparisons. Values were expressed as mean ± SD and P < 0.05 was considered statistically different. ^#^ P < 0.05 compared to SC with SGBS medium and *:P < 0.05 compared to SC with Lonza medium.

The composition of the differentiation medium can impact on adipocyte differentiation and thus we examined effect of indomethacin and rosiglitazone during the early phase of our experiments. UCP1 gene expression remained similar in SGBS and PHWSCs when differentiated with medium that is optimal for either cell types (expressed as Dct, Fig. 1). To maintain PHWSC viability while differentiating with SGBS differentiation medium, we supplemented the medium with 10% FBS which is commonly used in adipocyte differentiation cocktails for primary human adipocytes^12^.

**Figure 2:**
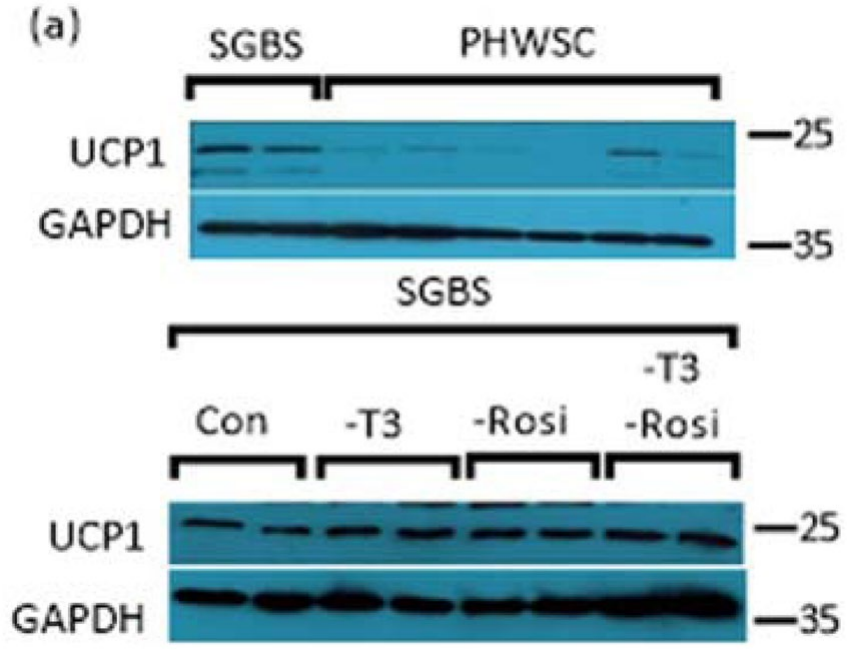
UCP1 protein expression in SGBS and PHWSC (upper panel). In an independent experiment, SGBS were differentiated in T3 and rosiglitazone depleted medium (lower panel).

In results obtained by Tews *et al*., UCP1 was higher expressed in adipocytes differentiated with rosiglitazone compared to indomethacin, where UCP1 expression was hardly detectable. SGBS cells showed stronger UCP1 expression compared to hASCs when differentiated with rosiglitazone. Interestingly, in our experiments UCP1 expression remained detectable and the amount of UCP1 were similar between control and the ones without rosiglitazone when we omitted rosiglitazone (Fig. 2). Further studies will be needed to explain these observed differences.

### Technical considerations

In our results obtained from SGBS adipocytes, isoproterenol (ISO) treatment led to a sharp rise in OCR and as expected, introduction of (fatty acid free-bovine serum albumin) FAF-BSA diminished the effect of ISO in a dose dependent manner. ISO-induced leak respiration, which is considered a better representation of UCP1 activity, was nearly two folds higher than basal leak respiration in absence of FAF-BSA. As a confirmative marker of browning in cells using the Agilent Seahorse flux analyzer, it is now commonly recommended to measure OCR after stimulating cells with lipolytic stimuli combined with the presence or absence of BSA in the medium, since it measures the increase in OCR due to free fatty acid metabolism derived from cellular lipolysis. However, we acknowledge that the oligomycin-protonophore based assay used here is not specific to UCP1- dependent proton leak. This is a technical limitation, and future studies that assess direct measures of UCP1 function should take this into consideration.

Another consideration to note is that the absorbance obtained from Janus Kinase B method is dependent on the number of mitochondria in the cell. The number of cells obtained from Janus Kinase B method is accurate for a broad range of cell numbers, but the methodology needs to be optimised for each cell type before absorbance can be considered directly proportional to the cell number^15^. In any given experimental setup, using the absolute absorbance to normalize cell numbers in SeaHorse experiments may not be ideal as differences in the number of mitochondria per cell may mask variation in OCR observed in adipocytes. Alternative methods include nuclear staining such as DAPI or Hoechst or using total protein concentration as a basis for normalizing cell numbers.

Overall, key differences in the (i) clinical characteristics of the donor from which the cells were derived from, (ii) adipose tissue depot from which samples were collected from, (iii) composition of cell culture media, and (iv) approaches to the analyses of functional readouts can explain the phenotypic differences observed in primary human adipocytes, when comparing findings from both studies. In our opinion, both manuscripts provide valuable insights into how these differences can affect the resultant characteristics observed from different human cells lines, and underscores the importance of understanding the strength and limitations of different human derived in vitro models and the conditions used. Applying these considerations can then guide the selection of appropriate models and the conditions used, to optimally address specific research questions of interest.

## Data Availability

All data generated or analysed during this study are included in this published article.
